# The Anti-Oxidant Defense System of the Marine Polar Ciliate *Euplotes nobilii*: Characterization of the *MsrB* Gene Family

**DOI:** 10.3390/biology6010004

**Published:** 2017-01-18

**Authors:** Francesca Ricci, Federico M. Lauro, Joseph J. Grzymski, Robert Read, Rigers Bakiu, Gianfranco Santovito, Pierangelo Luporini, Adriana Vallesi

**Affiliations:** 1 School of Biosciences and Veterinary Medicine, University of Camerino, Camerino 62032, Italy; francesca.ricci@unicam.it (F.R.); piero.luporini@unicam.it (P.L.); 2 Singapore Centre for Environmental Life Sciences Engineering (SCELSE), Nanyang Technological University, 60 Nanyang Drive, SBS-01N-27, Singapore 637551, Singapore; flauro@ntu.edu.sg; 3 Division of Earth and Ecosystem Sciences, Desert Research Institute, Reno, NV 89512, USA; Joe.Grzymski@dri.edu (J.J.G.); Robert.Read@dri.edu (R.R.); 4 Department of Aquaculture and Fisheries, Agricultural University of Tirana, Tirana 1019, Albania; rigers.bakiu@ubt.edu.al; 5 Department of Biology, University of Padova, via U. Bassi 58/B, Padua 35100, Italy; gianfranco.santovito@unipd.it

**Keywords:** methionine sulfoxide reductase, MsrB proteins, oxidative stress, polar microbiology, ciliate nano-chromosomes, *Euplotes*

## Abstract

Organisms living in polar waters must cope with an extremely stressful environment dominated by freezing temperatures, high oxygen concentrations and UV radiation. To shed light on the genetic mechanisms on which the polar marine ciliate, *Euplotes nobilii*, relies to effectively cope with the oxidative stress, attention was focused on methionine sulfoxide reductases which repair proteins with oxidized methionines. A family of four structurally distinct *MsrB* genes, encoding enzymes specific for the reduction of the methionine-sulfoxide R-forms, were identified from a draft of the *E. nobilii* transcriptionally active (macronuclear) genome. The *En-MsrB* genes are constitutively expressed to synthesize proteins markedly different in amino acid sequence, number of CXXC motifs for zinc-ion binding, and presence/absence of a cysteine residue specific for the mechanism of enzyme regeneration. The En-MsrB proteins take different localizations in the nucleus, mitochondria, cytosol and endoplasmic reticulum, ensuring a pervasive protection of all the major subcellular compartments from the oxidative damage. These observations have suggested to regard the *En-MsrB* gene activity as playing a central role in the genetic mechanism that enables *E. nobilii* and ciliates in general to live in the polar environment.

## 1. Introduction

Among the abiotic stresses that affect the life in polar coastal seawaters, high oxygen concentrations and enhanced UV radiations play a central role. They are general causes of increased production of reactive oxygen species (ROS). At protein level, methionine is one of the most oxidation-sensitive amino acids [1,2,3], and its oxidation by ROS to Met-sulfoxide results in the formation of two, *R* and *S*, stereoisomers at the sulfur atom. Organisms repair their methionine-oxidized proteins through the enzymatic activity of two structurally distinct classes of methionine sulfoxide reductases, designated as MsrA (EC 1.8.4.11) and MsrB (EC 1.8.4.12). The former catalyzes the reduction of the methionine-*S*-sulfoxide (Met-*S*-SO), and the latter catalyzes the reduction of the methionine-*R*-sulfoxide (Met-*R*-SO) [4].

In a previous comparative study between two phylogenetically closely allied ciliate species, namely *Euplotes nobilii* which is distributed in both Antarctic and Arctic coastal waters [5,6,7,8] and *E. raikovi* dwelling in temperate mid-latitude seas [9], it was observed that *E. nobilii* not only recovers more quickly from UV damages, but also withstands much more effectively noxious concentrations of hydrogen peroxide [10]. The implication relevant to these observations that the *E. nobilii* adaptation to the polar environment involves a particularly potent activity of its antioxidant genes is supported here by showing that it constitutively expresses a family of four distinct *MsrB* genes, each encoding a protein that takes a distinct localization within the cell. One of these genes was previously identified by PCR amplification and cloning of DNA preparations [11]. The three other genes have now been identified by an in silico analysis of a draft assembly of the transcriptionally active, sub-chromosomic genome that resides in the cell somatic nucleus (macronucleus) and includes only gene-size DNA molecules (or nano-chromosomes) each amplified to hundred or even thousand copies [12].

## 2. Materials and Methods

### 2.1. Cells

The *E. nobilii* wild-type strain 4Pyrm4, isolated from a coastal site at Pyramiden (Svalbard Islands) [7,8], was used. It was cultivated in a cold room, at 4–6 °C, with a daily cycle of 12 h of dark and 12 h of very weak light. The green alga *Dunaliella tertiolecta* was the standard food source.

### 2.2. DNA Isolation and Sequencing

Preparations of DNA were obtained from cell cultures according to standard protocols [13], and 10-μg aliquots were used to construct libraries of 250 bp-inserts. These libraries were sequenced by a HiSeq 2000 Illumina platform (Illumina Inc, San Diego, CA, USA) and assembled de novo using Trinity in paired-end mode, with a maximal memory usage of 10 Gb [14]. The assembly generated 37,420 contigs with an N50 score of 2333 bp for a total of 57,810,252 bp. The total GC content of the assembly was 38.07%. To contain the generated contigs, a custom BLAST database was created according to Altschul et al. [15].

### 2.3. RNA Extraction and Gene Expression Analysis

Total RNA was extracted from cell cultures using the TRIzol plus RNA purification kit (Life Technologies, Carlsbad, CA, USA). To avoid DNA contamination, a DNase-digestion step was performed during RNA purification, according to the on-column DNase treatment protocol (Life Technologies).

For the cDNA synthesis, 2 μg-aliquots of total RNA were reverse-transcribed with the Super Script III Reverse Transcriptase (Life Technologies) in a 20-μL volume, using oligo(dT)-AP (Table 1) or random hexamers to start the reaction in the presence of 40 units of RiboLock (Thermo Fisher Scientific, Waltham, MA, USA), as recommended by the manufacturer (Life Technologies). Aliquots (1 μL) of each reaction were directly used as templates for PCR amplifications, which were run in an Eppendorf Ep-gradient Mastercycler (Eppendorf AG, Hamburg, Germany) with oligonucleotides synthesized by Invitrogen (Life Technologies) as primers (Table 1). Amplification cycles consisted of a 94 °C denaturation step for 30 s, a 58 °C annealing step for 30 s, and a 72 °C elongation step for 30 s. For each primer combination, the products of two separate amplifications were purified using the NucleoSpin PCR clean-up kit (Macherey-Nagel GmbH, Duren, Germany) and sequenced at the BMR Genomics Centre (Padua, Italy).

For semi-quantitative PCR analysis (sqRT-PCR), 1 μL-aliquots of cDNA were amplified using gene specific primers (Table 1) designed to obtain products of 200–250 bp. A negative control containing RNA, instead of cDNA, was run in parallel. Thirty-five PCR cycles were run to reach the exponential phase of cDNA amplification. Equivalent volumes of amplification products were separated by gel electrophoresis, visualized by ethidium bromide staining and quantified using the ImageJ software (NIH, Bethesda, MD, USA). Amplicons of 18S rRNA were used to normalize the levels of gene expression.

### 2.4. Sequence Analysis and Phylogenetic Relationships

The nucleotide gene sequences and the functional domains of the deduced amino acid sequences were analyzed with the SECISearch tool (http://genome.unl.edu/SECISearch.html) and ScanProsite (http://prosite.expasy.org/), respectively. The amino acid sequences used to establish the En-MsrB phylogenetic relationships were aligned with the T-Coffee multiple sequence alignment package [16]. The statistical selection of the best-fit models was carried out with ProtTest 3 [17]. The phylogenetic relationships were assessed using 122 candidate models, and adopting the ‘Bayesian Information’ (BI) criterion along with the ‘Akaike Information’ criterion used also in the corrected version. The best-fit (clock, no-clock, or relaxed clock) model was selected as previously described [18], by assessing the marginal model likelihoods by means of the stepping-stone method that samples a series of distributions representing different mixtures of the posterior and prior distributions [19]. It was applied to the dataset of the MsrB amino acid sequences using 510,000 generations with a diagnostic frequency of 2,500 in two independent runs for each model.

The phylogenetic tree was built with the BI method implemented in MrBayes 3.2 (Department of Biodiversity Informatics, Swedish Museum of Natural History, Stockholm, Sweden) [20], and displayed with the FigTree v1.3 software (Institute of Evolutionary Biology, Edinburgh, UK). Four independent runs, each with four simultaneous ‘Markov Chain Monte Carlo’ chains, were performed for 10^6^ generations and sampled every 10^3^. In applying the maximum likelihood (ML) method implemented in PhyML 3.0 (Méthodes et Algorithmes pour la Bioinformatique, LIRMM, Centre National de la Recherche Scientifique, Université de Montpellier, Montpellier, France) [21], bootstrap analyses were performed on 10^4^ trees and the tree topology was improved with both the ‘Nearest Neighbor Interchange’ and the ‘Subtree Pruning and Re-grafting’.

## 3. Results

### 3.1. Gene Identification and Nucleotide Sequences

**Figure 1:**
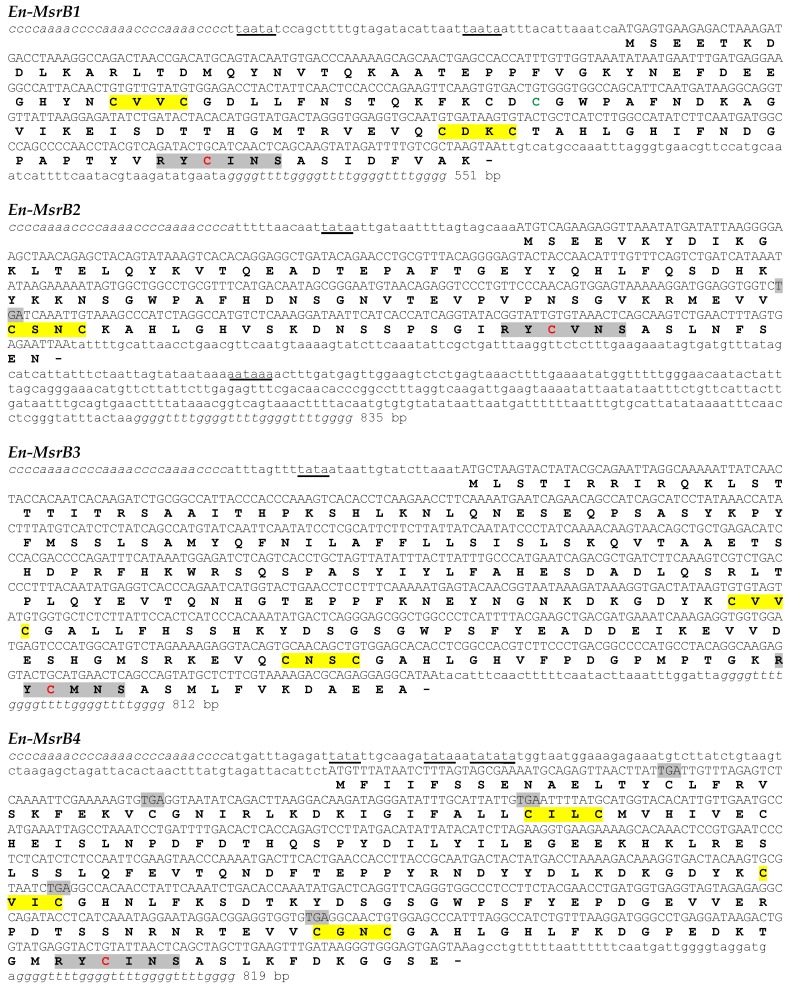
Nucleotide sequences of the *En-MsrB* genes and deduced amino acid sequences. In the nucleotide sequences, the telomeric repeats are in italics, the 5’ leader and 3’ trailer regions in lower case letters and the open reading frame in capital letters. The putative transcriptional regulation and polyadenylation signals are underlined, and the in-frame TGA codons highlighted in grey. In the amino acid sequences, the CXXC motifs and the catalytic sites are highlighted in yellow and grey, respectively, and the catalytic and the resolving cysteines are highlighted in red and green, respectively.

The amino acid sequence of the previously identified *E. nobilii* MsrB protein [11], hereafter designated as En-MsrB1, was used as query to search for new genes encoding Msr of type B on the BLAST database of a draft of the *E. nobilii* macronuclear genome. In addition to the *En-MsrB1* gene, three new genes were identified and designated *En-MsrB2*, *En-MsrB3* and *En-MsrB4* (Figure 1).

The four *En-MsrB* genes have the typical organization of the *Euplotes* macronuclear genes, containing a single open reading frame (ORF) flanked with 5’ leader and 3’ trailer non-coding regions capped with telomeric C_4_A_4_ and G_4_T_4_ repetitions. They are characterized by a 5’ leader region which is rather uniform in extension and rich in A and T repetitions arranged to form canonical TATA boxes and less common TAATA motifs (in the *En-MsrB1* gene) specific for the transcription regulation. In contrast, the 3’ trailer region is extremely variable in length (from only 36 bp in the *En-MsrB3* gene to 404 bp in the *En-MsrB2* gene) and devoid of canonical AATAAA polyadenylation signals for the termination of transcription, except that in the *En-MsrB2* gene which contains one such signal. The ORF is identified with the first ATG start codon, except that in the *En-MsrB4* gene which includes TAA or TAG stop codons immediately downstream of the first five ATG codons. The ORF of this gene thus coincides with the sixth ATG.

In-frame TGA stop codons distinguish the ORF of the *En-MsrB2* and *En-MsrB4* genes. However, they do not act as effective stop signals because *Euplotes* uses the TGA codon to specify cysteine [22], or even selenocysteine [23]. The co-translational incorporation of this amino acid into the nascent protein chain is directed by specific mRNA secondary structures designated selenocysteine insertion sequence (SECIS) elements, which are responsible for the mRNA binding to selenocysteine-specific translation factors [24]. The *En-MsrB2* and *En-MsrB4* genes lack any SECIS element, which excludes a selenoprotein nature of their products.

### 3.2. Amino Acid Sequences

**Figure 2:**
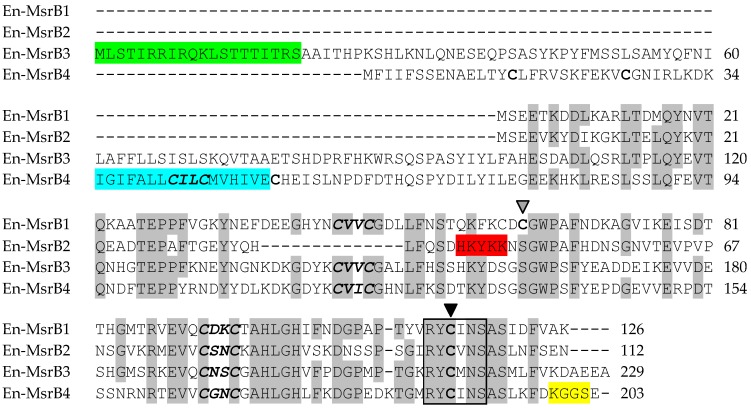
Amino acid sequence alignment of the En-MsrB proteins. The alignment was maximized by gap insertion and residues identical in three or four sequences are highlighted in grey. The CXXC motifs are in bold and italics. The resolving and catalytic cysteines are indicated by grey and filled arrowheads, respectively. The conserved catalytic site is boxed. Signals for mitochondrial targeting, nuclear translocation, transmembrane domain and ER retention are highlighted in green, red, blue and yellow, respectively.

The proteins (En-MsrB1 to En-MsrB4) encoded by the four *En-MsrB* genes markedly vary from one another in chain length (112 amino acid residues in En-MsrB2, 229 in En-MsrB3) and degree of sequence similarity (42.6% identity in 122 residue-overlaps between En-MsrB2 and En-MsrB3, 56.8% identity in 139 residue-overlaps between En-MsrB3 and En-MsrB4) (Figure 2). However, in spite of these variations, they all uniformly show the MsrB-specific ‘catalytic Cys’ lying within a highly conserved Arg-Tyr-Cys-Met/Val/Ile-Asn-Ser-Ala-Ser sequence segment of the carboxyl-terminal region.

According to the TargetP (Center for Biological Sequence Analysis, Technical University of Denmark, Lyngby, Denmark) and PSORT II softwares (Human Genome Center, Institute for Medical Science, University ot Tokyo, Japan) [25,26], each En-MsrB protein takes an its own specific subcellular localization: (i) the cytoplasm for En-MsrB1, which lacks any signal-peptide motif necessary for the protein transport across membranes; (ii) the nucleus for En-MsrB2, in which the motif His_43_-Lys-Tyr-Lys-Lys_47_ (canonically formed by a repetition of basic amino acids) forms a mono-partite nuclear localization signal [27]; (iii) mitochondria for En-MsrB3, in which the first 20 amino acids of the amino-terminal region form a mitochondrial signal sequence; and (iv) the endoplasmic reticulum (ER) for En-MsrB4 which, in addition to including a putative transmembrane domain (positions 35–51) inside its amino-terminal region, also possesses a ER-specific retention signal provided by the Lys_199_-Gly-Gly-Ser_202_ motif in the carboxyl terminal region (Figure 2).

### 3.3. Phylogenetic Relationships

**Figure 3:**
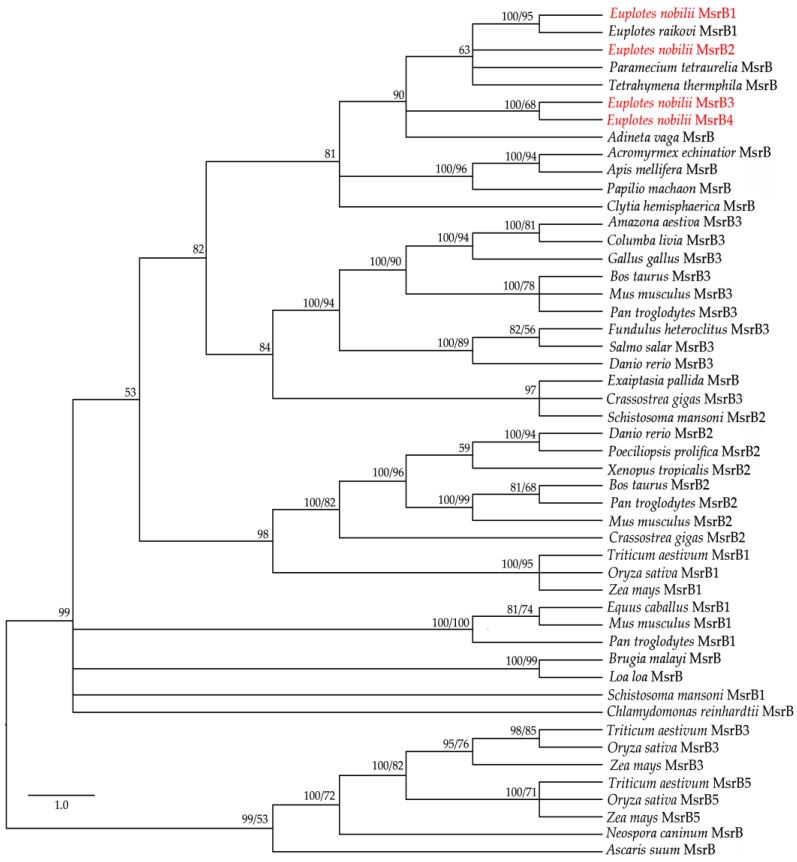
Phylogenetic relationships of the En-MsrB proteins (in red) with MsrBs from other organisms. Tree reconstruction based on both BI and ML methods. Posterior probability (first number) and boostrap values (second number, when present) are indicated at the nodes. The scale for the branch length is shown on the left.

The application of the BI and ML methods to a selected panel of eukaryotic MsrB amino acid sequences available from the GenBank database (MsrBs’ accession numbers can be found in Appendix, Table A1) generated closely similar trees (Figure 3), in which the four En-MsrBs group together into a statistically well supported clade including MsrB sequences of other ciliates and a MsrB sequence described as of non-metazoan origin from the bdelloid rotifer, *Adineta vaga* [28]. Within this clade, En-MsrB1 finds its counterpart with a MsrB sequence from *E. raikovi*, while En-MsrB2 associates with MsrBs from *Paramecium* and *Tetrahymena*, and En-MsrB4 and En-MsrB4 form their own branch.

### 3.4. Gene Expression

**Figure 4:**
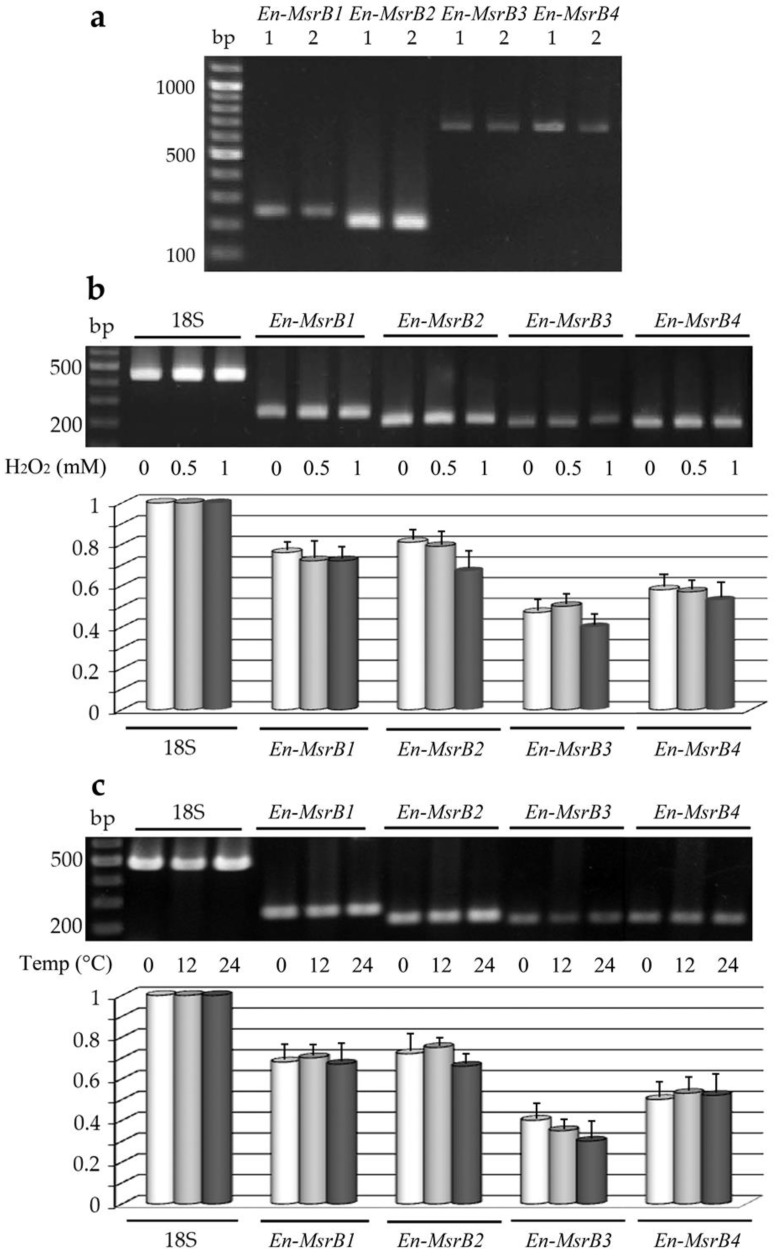
Expression of the *E. nobilii En-MsrB* genes. (**a**) Gel electrophoresis of PCR products obtained by amplifying DNA (lanes 1) or cDNA (lanes 2) preparations with *En-MsrB* gene-specific primers. (**b**) Electrophoretic separation (upper panel) and relative transcript abundance (lower panel) of sqRT-PCR products obtained from cells treated for 30 min with increasing concentrations of H_2_O_2_. (**c**) Electrophoretic separation (upper panel) and relative transcript abundance (lower panel) of sqRT-PCR products obtained from cells exposed for 30 min at increasing temperatures. In both (**b**) and (**v**), values were calculated taking the 18S-rRNA PCR fragments as value 1 and represent the means (± SD) of three independent experiments.

First, we analyzed whether each *En-MsrB* gene is effectively expressed by preparing cDNA from RNA extracted from growing cells, and amplifying aliquots of cDNA via PCR with primer combinations specific to sequence segments adjacent to the extremities of each gene ORF (Table 1). Amplicons of each gene were obtained and directly sequenced (Figure 4a). No intron was found to interrupt the ORF sequence of the four genes. Also, no intron sequence was identified within the exceptionally long (404 bp) 3’ trailer region distinctive of the *En-MsrB2* gene. Evidence for this conclusion was obtained by amplifying cDNA preparations with a forward primer specific to a sequence internal to the *En-MsrB2* ORF and a reverse primer provided by the same oligo (dT)-AP used for cDNA synthesis, and observing that the 3’ untranslated region of each transcript fully matched the uninterrupted 134-bp gene sequence comprised between the TAA stop codon of the ORF and the G nucleotide located 9-bp downstream the AATAAA polyadenylation signal.

Second, we analyzed whether the expression of each *En-MsrB* gene can be induced to increase in cells exposed to oxidative and thermal stress conditions. The gene expression in response to the oxidative stress was analyzed in cells suspended for 30 min in fresh seawater with H_2_O_2_ added in increasing concentrations. The gene expression in response to the thermal stress was analyzed in cells suspended for 30 min at their standard cultivation temperature of 4 °C and at increased temperatures of 12 and 24 °C. Preparations of cDNA obtained from each cell suspension were amplified by sqRT-PCR and the relative amounts of the amplified gene transcripts were compared between stressed and not-stressed cells (Figure 4b,c). The *En-MsrB* genes revealed different levels of expression, with the *En-MsrB3* gene systematically characterized by the production of appreciably lower amounts of transcript. However, no *En-MsrB* genes showed a significant quantitative difference in the transcript abundance between stressed and not-stressed cells implying that they behave as constitutive genes, each transcribed at a relatively constant level.

## 4. Discussion

Like numerous other eukaryotic organisms, *E. nobilii* relies on the activity of multiple *En-MsrB* genes to repair Met-oxidized proteins. However, while in animals and plants the expression of these genes is generally responsive to environmental change [29,30], in *E. nobilii* the expression of all four *En-MsrB* genes has been found to remain substantially unchanged in cells exposed to oxidative and thermal stresses. Consistently with their constitutive expression, the *En-MsrB* genes have a short 5’ leader non-coding region devoid of *cis*-active enhancer motifs directly involved in up-regulating the genetic response in stressed organisms, such as the CCCCT ‘stress-response element’ of *Saccharomyces cerevisiae* [31], the TGAG/CNNNGC ‘antioxidant responsive elements’ of mammalian cells [32], and the TGACNNN ‘half antioxidant response elements’ that are common in other organisms including the ciliate *Tetrahymena thermophila* [18,33,34].

The constitutive expression of the *En-MsrB* genes is likely fundamental for the efficiency of the *E. nobilii* physiological mechanism of ROS scavenging, that in oxidizing environments has been proposed to be constantly activated by a methionine oxidation/reduction cycling [35]. However, lack of appropriate information on the constitutive, or inducible nature of *Msr* genes in other *Euplotes* species suggests caution in concluding that the constitutive expression of the *En-MsrB* genes reflects a specific adaptive trait of *E. nobilii* the to the stressful conditions of the polar environment. Only in *E. raikovi* two *Msr* genes have been identified and initially analyzed for their expression. One gene encoding a protein structurally related to En-MsrB1 is constitutively expressed like the *En-MsrB* genes [11]. The other gene encoding a MsrA protein seems to be an inducible gene [10]. However, it does not appear to be endogenous to *E. raikovi*. It was supposed to derive from Alphaproteobacteria through a phenomenon of lateral gene transfer [36].

**Figure 5:**
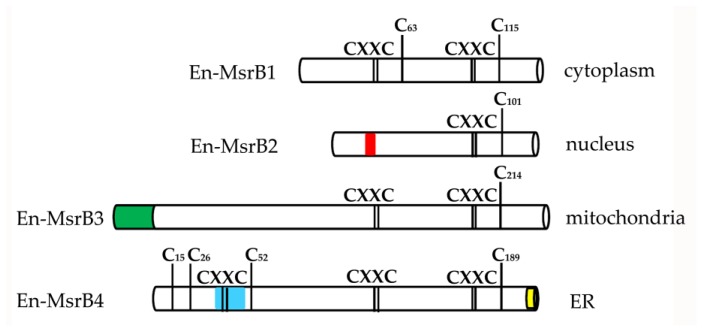
Schematic representation of the En-MsrB protein structure. The positions of the zinc-ion binding CXXC motifs, the catalytic and resolving cysteines, and the additional Cys residues specific of the En-MsrB4 amino-terminal region are indicated by bars. Boxes of different color indicate the relative positions and length of the signals for mitochondrial targeting (green), ER retention (yellow), nuclear localization (red) and trans-membrane domain (blue).

The proteins synthesized by the four *En-MsrB* genes showed remarkably diversified amino acid sequences. The functionally more important variations reside in the number of the CXXC motifs that cooperate for the binding of the zinc ion, and the presence/absence and localization of a Cys residue, known as ‘resolving cysteine’, that is involved in the mechanism of MsrB regeneration (Figure 5). En-MsrB1 and En-MsrB3 are the only two proteins that, like many other eukaryotic MsrBs, orthodoxically contain two CXXC motifs for the zinc-ion binding (i.e., Cys_45_-Val-Val-Cys_48_ and Cys_92_-Asp-Lys-Cys_95_ in En-MsrB1; Cys_144_-Val-Val-Cys_147_ and Cys_191_-Asn-Ser-Cys_194_ in En-MsrB3). A single CXXC motif (i.e., Cys_78_-Ser-Asn-Cys_81_) is distinctive of En-MsrB2 which, as a consequence, should be unable to bind the zinc ion. The crucial role that this ion plays in the stabilization of the MsrB molecular structure [37,38,39], thus suggests that En-MsrB2 may rely on an increased structural flexibility to interact more effectively with carrier proteins on the way to its subcellular nuclear localization. En-MsrB4 is another structurally rather eccentric MsrB protein because it includes a third CXXC motif (i.e., Cys_42_-Ile-Leu-Cys_45_) in the amino terminal region. The role, if any, played by this additional motif remains enigmatic.

With regard to the presence/absence of the resolving cysteine, En-MsrB1 is unique because it includes both this cysteine (Cys_63_) and the catalytic one (Cys_115_) in the carboxyl-terminal region. This inclusion makes it a member of the so-called ‘2-Cys MsrBs’, in which the resolving and catalytic cysteines form an intra-chain disulfide bridge destined to be reduced by thioredoxin through disulfide exchange [40,41,42]. Instead, all other En-MsrBs are characterized by the replacement of the canonical resolving cysteine with a Ser residue. However, while in the recycling process En-MsrB4 may use one of the three additional Cys residues included in its amino-terminal region as resolving cysteine, En-MsrB2 and En-MsrB3 do not possess any additional Cys residue to replace the loss of the canonical resolving cysteine. These two proteins thus belong to the group of the so-called ‘1-Cys MsrBs’, whose regeneration avoids the formation of an intra-chain disulfide bond and involves two alternative mechanisms. In one mechanism, glutathione is the reductant of the sulfenic acid formed on the catalytic cysteine after the substrate reduction, and the deglutathionylation step is then achieved by the activity of glutaredoxins [43]. In the second mechanism, thioredoxins directly interact with oxidized 1-Cys MsrBs and regenerate their activity without the help of any other thiol compound [44,45]. Which of the two mechanisms is used by *E. nobilii* to regenerate its En-MsrB2 and En-MsrB3 proteins remains to be investigated.

In addition to the four *En-MsrB* genes, the scanning of the *E. nobilii* genome identified a fifth gene encoding a ‘MsrB-like’ protein and at least three gene sequences encoding Msr enzymes of type A. Although the MsrB-like protein contains a zinc-ion binding site represented by a double CXXC motif, it lacks the MsrB-specific catalytic cysteine which has been replaced with a Thr residue. Because of this substitution it thus more closely recalls the structure of the ‘SelR’ proteins with unknown function of *Oxytricha*, *Stylonychia*, *Paramecium* and *Tetrahymena* which similarly possess a double CXXC motif and a canonical catalytic cysteine replaced with a Ser residue.

With regard to the MsrA-coding gene sequences, we can anticipate that they show an origin which is clearly not endogenous to *E. nobilii*, because they lack the telomeric nucleotide C_4_A_4_ repeats that are the distinctive structural marker of every *Euplotes* macronuclear gene [46]. Instead, they unmistakably cluster close to sequences of prokaryotic *MsrA* genes, in particular to *MsrA* gene sequences that are specific of species of the bacterium *Francisella*. These bacteria live as common and pervasive cytoplasmic endosymbionts of *Euplotes* species [47,48], suggesting that *E. nobilii* demands the reduction of its methionine-sulfoxide S-forms to the exogenous enzymatic activity of its bacterial endosymbionts.

## 5. Conclusions

The four structurally distinct anti-oxidant *MsrB* genes that have been identified in the somatic (macronuclear) genome of the polar ciliate *E. nobilii* behave as constitutive genes, in contrast with the inducible response that is commonly shown by *Msr* genes in other organisms. Their products are markedly diversified in the number and location of the Cys residues involved in the regeneration and zinc-ion binding mechanisms, and include sequence domains specific to localize and be active in distinct subcellular compartments.

## Figures and Tables

**Table 1:** Primer combinations used in PCR amplifications and relative dimensions of PCR products.

Primer Combination	Primer Sequences (5’–3’)	Product Size (bp)
B1FW + B1RV	AAAGGCCAGACTAACCGACA + CATTGCACCTCCACCCTAGT	249
B2FW + B2RV	AAAGTCACACAGGAGGCTGA + CATGGCCTAGATGGGCTTTA	208
B2FW + oligo(dT)-AP	AAAGTCACACAGGAGGCTGA + GGCCACGCGTCGACTAGTACT_17_	480
B3FW + B3RV	ATGCTAAGTACTATACGCAGAA + TATGCCTCCTCTGCGTCTTT	689
B3FW2 + B3RV	CTGGCCCTCATTTTACGAAG + TATGCCTCCTCTGCGTCTTT	201
B4FW + B4RV	ATGGTAATGGAAAGAGAAATG + ACTCACTCCCACCCTTATCA	686
B4FW2 + B4RV	GTGGCCCTCCTTCTACGAAC + ACTCACTCCCACCCTTATCA	200

## Appendix

**Table A1:** MsrB proteins included in the phylogenetic analysis: Source, form and GenBank accession number.

Species	Form	Accession Number
*Euplotes nobilii*	B1	AFZ61876.1
*Euplotes nobilii*	B2	KY311562 ^1^
*Euplotes nobilii*	B3	KY311563 ^1^
*Euplotes nobilii*	B4	KY311564 ^1^
*Acromyrmex echinatior*	B	GL888413.1
*Adineta vaga*	B	EU637017.1
*Amazona aestiva*	B3	LMAW01002576.1
*Apis mellifera*	B	XM_003251284.3
*Ascaris suum*	B	JI212431.1
*Bos taurus*	B2	GJ062270.1
*Bos taurus*	B3	GJ060799.1
*Brugia malayi*	B	XM_001900358.1
*Chlamydomonas reinhardtii*	B	EDP03924.1
*Clytia hemisphaerica*	B	GBGP01000067.1
*Columba livia*	B3	KB375322.1
*Crassostrea gigas*	B2	JH821652.1
*Crassostrea gigas*	B3	JH816866.1
*Danio rerio*	B2	NM_212921.2
*Danio rerio*	B3	BC071530.1
*Equus caballus*	B1	NM_001170424.1
*Euplotes raikovi*	B1	JX978448.1
*Exaiptasia pallida*	B	LJWW01000100.1
*Fundulus heteroclitus*	B3	GCES01009679.1
*Gallus gallus*	B3	NM_001199578.1
*Loa loa*	B	JH712120.1
*Mus musculus*	B1	NM_013759.2
*Mus musculus*	B2	BC021619.1
*Mus musculus*	B3	NM_177092.4
*Neospora caninum*	B	LN714486.1
*Oryza sativa*	B1	LOC_Os06g2776
*Oryza sativa*	B3	LOC_Os05g33510
*Oryza sativa*	B5	LOC_Os03g24600
*Pan troglodytes*	B1	NM_001114751.1
*Pan troglodytes*	B2	GABE01001715.1
*Pan troglodytes*	B3	GABE01000482.1
*Papilio machaon*	B	XM_014504817.1
*Paramecium tetraurelia*	B	XP_001426263
*Poeciliopsis prolifica*	B2	GBYX01192342.1
*Salmo salar*	B3	XM_014153858.1
*Schistosoma mansoni*	B1	AY669150.1
*Schistosoma mansoni*	B2	AY669149.1
*Tetrahymena thermophila*	B	XP_001019714.4
*Triticum aestivum*	B1	KP030665
*Triticum aestivum*	B3	KP030667
*Triticum aestivum*	B5	KP030670
*Zea mays*	B1	GRMZM2G025322
*Zea mays*	B3	GRMZM2G089308
*Zea mays*	B5	GRMZM2G577677
*Xenopus tropicalis*	B2	BC171252.1
*Paramecium tetraurelia*	B	XP_001426263

^1^ This work.
